# P-1093. In Vitro Activity of Eravacycline against Carbapenem-Resistant *Acinetobacter baumannii* as Evaluated by Broth Microdilution, MIC Test Strip and Disc Diffusion

**DOI:** 10.1093/ofid/ofae631.1281

**Published:** 2025-01-29

**Authors:** Qihui Liu, Shirong Li, Haoqin Jiang, Xuan Wang, Ning Li

**Affiliations:** Huashan Hospital, Shanghai Medical College, Fudan University, Shanghai, Shanghai, China (People's Republic); Huashan Hospital, Shanghai Medical College, Fudan University, shanghai, Shanghai, China; Huashan Hospital, Shanghai Medical College, Fudan University, Shanghai, Shanghai, China (People's Republic); Huashan Hospital, Shanghai Medical College, Fudan University, Shanghai, Shanghai, China (People's Republic); Huashan Hospital, Shanghai Medical College, Fudan University, Shanghai, Shanghai, China (People's Republic)

## Abstract

**Background:**

Eravacycline is a new type of fully synthetic fluorocycline antibiotic. The Chinese Committee on Antimicrobial Susceptibility Testing (ChinaCAST) published the clinical breakpoints of eravacycline, including MIC breakpoints and their inhibition zone diameter correlates. MIC test strip (MTS) is a gradient diffusion method that represents a simple alternative to the broth microdilution (BMD) method for performing antimicrobial susceptibility testing (AST). The aim of this study was to compare the susceptible rate of carbapenem-resistant *Acinetobacter baumannii* (CRAB) to eravacycline by three different testing methods.

**Figure:**
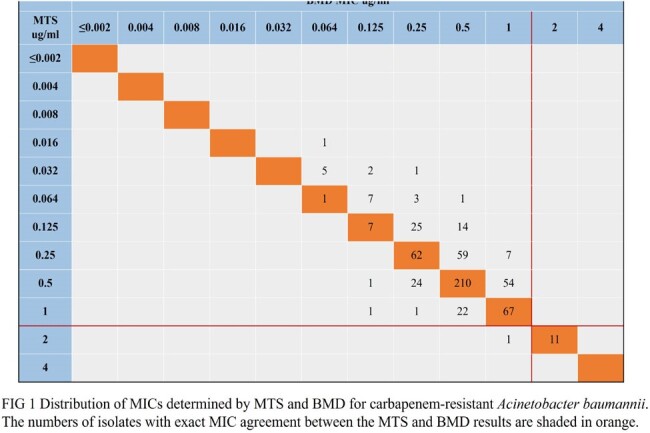

**Methods:**

587 strains of CRAB were collected from the Huashan Hospital affiliated to Fudan University from 2019 to 2023. According to the antimicrobial susceptibility test recommended by the American Society for Clinical and Laboratory Standards 2023, the activity of eravacycline was determined. In this study, the BMD was used as the reference standard, and the clinical breakpoints standard published by ChinaCAST ( *Acinetobacter baumannii* susceptible MIC, ≤ 1 μg / mL or inhibition zone diameter , ≥ 15 mm ) was used to detect the susceptible rate of eravacycline to CRAB.

**Figure:**
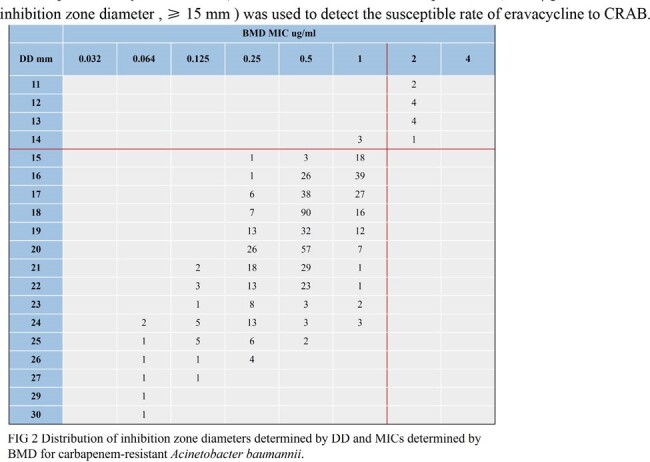

**Results:**

The MIC_50/90_s were as follows: BMD, 0.5/1 mg/L; MTS, 0.38/0.75 mg/L. Based on the BMD method, the susceptible rate of CRAB to eravacycline was 98.13% (576/587) according to the ChinaCAST breakpoints, while 97.96% (575/587) was observed in MTS method. The results of DD method showed that the sensitivity rate was 97.61% (573/587). Compared with the results of BMD, the essential agreement (EA) rate of MTS was 94.55%. The categorical agreement (CA) rates of MTS and DD were 99.83% and 99.49%, respectively. The total major error (ME) rate and total very major error (VME) rate of MTS were 0.17% and 0%, respectively. The total ME rate and total VME rate of DD were 0.51% and 0%, respectively.

**Conclusion:**

The susceptible rate of eravacycline to CRAB is high. CA, ME or VME all met the methodological evaluation indicators. The results of the sensitivity of CRAB to eravacycline determined by MTS and DD methods were similar, and the consistency with the BMD method was good. Both of them can be used as reliable AST methods for the determination of eravacycline antibacterial activity in clinical microbiology laboratories.

**Disclosures:**

**All Authors**: No reported disclosures

